# Development and validation of an ensemble machine-learning model for predicting early mortality among patients with bone metastases of hepatocellular carcinoma

**DOI:** 10.3389/fonc.2023.1144039

**Published:** 2023-02-20

**Authors:** Ze Long, Min Yi, Yong Qin, Qianwen Ye, Xiaotong Che, Shengjie Wang, Mingxing Lei

**Affiliations:** ^1^ Department of Orthopedics, The Second Xiangya Hospital of Central South University, Changsha, China; ^2^ Institute of Medical Information and Library, Chinese Academy of Medical Sciences and Peking Union Medical College, Beijing, China; ^3^ Department of Joint and Sports Medicine Surgery, The Second Affiliated Hospital of Harbin Medical University, Harbin, China; ^4^ Department of Oncology, Hainan Hospital of People's Liberation Army (PLA) General Hospital, Sanya, China; ^5^ Department of Evaluation Office, Hainan Cancer Hospital, Haikou, China; ^6^ Department of Orthopaedic Surgery, Shanghai Sixth People’s Hospital Affiliated to Shanghai Jiao Tong University, Shanghai, China; ^7^ Department of Orthopedic Surgery, Hainan Hospital of People's Liberation Army (PLA) General Hospital, Sanya, China; ^8^ Chinese People's Liberation Army (PLA) Medical School, Beijing, China

**Keywords:** bone metastases, machine learning, ensemble model, early mortality, hepatocellular carcinoma

## Abstract

**Purpose:**

Using an ensemble machine learning technique that incorporates the results of multiple machine learning algorithms, the study’s objective is to build a reliable model to predict the early mortality among hepatocellular carcinoma (HCC) patients with bone metastases.

**Methods:**

We extracted a cohort of 124,770 patients with a diagnosis of hepatocellular carcinoma from the Surveillance, Epidemiology, and End Results (SEER) program and enrolled a cohort of 1897 patients who were diagnosed as having bone metastases. Patients with a survival time of 3 months or less were considered to have had early death. To compare patients with and without early mortality, subgroup analysis was used. Patients were randomly divided into two groups: a training cohort (n = 1509, 80%) and an internal testing cohort (n = 388, 20%). In the training cohort, five machine learning techniques were employed to train and optimize models for predicting early mortality, and an ensemble machine learning technique was used to generate risk probability in a way of soft voting, and it was able to combine the results from the multiply machine learning algorithms. The study employed both internal and external validations, and the key performance indicators included the area under the receiver operating characteristic curve (AUROC), Brier score, and calibration curve. Patients from two tertiary hospitals were chosen as the external testing cohorts (n = 98). Feature importance and reclassification were both operated in the study.

**Results:**

The early mortality was 55.5% (1052/1897). Eleven clinical characteristics were included as input features of machine learning models: sex (p = 0.019), marital status (p = 0.004), tumor stage (p = 0.025), node stage (p = 0.001), fibrosis score (p = 0.040), AFP level (p = 0.032), tumor size (p = 0.001), lung metastases (p < 0.001), cancer-directed surgery (p < 0.001), radiation (p < 0.001), and chemotherapy (p < 0.001). Application of the ensemble model in the internal testing population yielded an AUROC of 0.779 (95% confidence interval [CI]: 0.727–0.820), which was the largest AUROC among all models. Additionally, the ensemble model (0.191) outperformed the other five machine learning models in terms of Brier score. In terms of decision curves, the ensemble model also showed favorable clinical usefulness. External validation showed similar results; with an AUROC of 0.764 and Brier score of 0.195, the prediction performance was further improved after revision of the model. Feature importance demonstrated that the top three most crucial features were chemotherapy, radiation, and lung metastases based on the ensemble model. Reclassification of patients revealed a substantial difference in the two risk groups’ actual probabilities of early mortality (74.38% vs. 31.35%, p < 0.001). Patients in the high-risk group had significantly shorter survival time than patients in the low-risk group (p < 0.001), according to the Kaplan–Meier survival curve.

**Conclusions:**

The ensemble machine learning model exhibits promising prediction performance for early mortality among HCC patients with bone metastases. With the aid of routinely accessible clinical characteristics, this model can be a trustworthy prognostic tool to predict the early death of those patients and facilitate clinical decision-making.

## Introduction

Primary liver cancer is the most frequent cause of cancer-related death in most regions of the world, and it is predicted to be the sixth most prevalent cancer worldwide in terms of incidence and mortality in 2020, with up to 906,000 new cases and 830,000 deaths (1). Hepatocellular carcinoma (HCC) is the most common type of liver cancer, and it accounted for 75% to 85% of all cases. Additionally, incidence and mortality are continually rising in many nations (2), and many HCC patients are still at an advanced stage when they are diagnosed (3). Viral hepatitis B and C and cirrhosis, fatty liver disease and diabetes, alcohol, and aflatoxin and aristolochic acid are among the main risk factors for HCC (3). Although the survival prognosis for HCC patients has improved significantly over the past 20 years, thanks to treatments, it is still unsatisfactory, with a median overall survival of only 16.5 to 16.2 months and a median progression-free survival of 5.6 to 5.7 months (4). Additionally, the 5-year survival rate remains less than 20% because of the high recurrence rate (5).

With the improvement of prognosis among HCC patients in recent years due to novel imaging techniques and multidisciplinary therapies, extrahepatic metastases now occur more frequently (6). The bone is a common extrahepatic metastatic site, and the prevalence ranged from 2.0% to 25.0% among patients with HCC (7, 8). Additionally, bone metastasis was responsible for 32.5% to 57.0% of all distant metastasis in HCC patients (9). HCC patients with bone metastases often had expansive soft tissue masses with severe osteolytic bone destruction and this may be explained by the theory of premetastatic niche (10, 11). Regarding prognosis, bone metastasis was a significant risk for survival outcome among HCC patients, and the median survival time was only 2.8–3.3 months among HCC patients with bone metastases (12, 13). The prognosis of those individuals may be improved by tailored therapy, and in order to implement individualized therapy, prediction models for evaluating the survival outcome among HCC patients with bone metastases must be developed.

A number of risk factors, including marital status (14), primary tumor surgery (14), Child-Pugh grade (15, 16), T stage (15), performance status, radiotherapy (17), the presence of ascites at the initial presentation (18), and the number of skeletal metastases (16), have been found to be significantly associated with the survival outcome of HCC patients with bone metastases. The establishment of survival prediction models for HCC patients with bone metastases is facilitated by these risk variables. Nevertheless, confounding factors that offer nonlinear influences and pose issues frequently have an impact on the survival prediction of patients with bone metastases. It should be noted that using machine learning techniques, this issue can be readily solved (19). Given the poor survival prognosis among those patients, short-term survival forecasting is crucial to create better plans and more appropriate responses. Therefore, this study aims to construct an accurate model to predict the early mortality (three-month mortality) among HCC patients with bone metastases using an ensemble machine learning technique that aggregated the results of multiple machine learning algorithms.

## Methods

### Data source and eligibility criteria

We extracted data from the Surveillance, Epidemiology, and End Results (SEER) Program. SEER is a large oncologic database which collects information on cancer diagnoses and survival for about 30% of the US population with the effort to reduce the cancer burden. We completed the registration form to obtain SEER*Stat (version 8.4.0.1) after reading and signing the Terms of Use Agreement. This software provides us with interface to access to the SEER database and download corresponding data.

Between January 1, 2000, and December 31, 2019, patients with histologically confirmed HCC were included for the analysis. The exclusive criteria were as follows (1): Patients did not have bone metastases (2); Patients younger than 18 years old (3); Patients did not have the histological diagnosis of adenomas and adenocarcinomas (4); Patients whose causes of death were missing or unknown (5); Patients were alive or dead of other reason (not attributable to liver cancer) with a follow-up interval of only three or less months; and (6) Patients whose survival time was unknown. Complete data were required for stage and liver cancer-specific mortality, and censoring was derived from the vital status recode.

All enrolled patients from the SEER database were divided into two groups: a model training cohort (n = 1509, 80%) and a model testing cohort (n = 388, 20%). The model testing cohort was regarded as the internal testing cohort, and the eligible patients from Hainan Hospital of Chinese PLA General Hospital (Sanya) and Hainan Cancer Hospital (Haikou) were served as the external testing cohort (n = 98). When users access to the SEER database, it is unnecessary to obtain formal ethics approval, since it is covered by its open access policy. This study was approved by the Hainan Hospital of Chinese PLA General Hospital and patients gave informed oral consent prior to data collection.

### Variable collection

Age, sex, race, marital status, tumor (T) stage, node (N) stage, fibrosis score, alpha fetoprotein (AFP) level, tumor size, brain metastases, liver metastases, lung metastases, surgery of lymph, cancer-directed surgery, radiation, and chemotherapy were all taken out of the SEER database. Patients having a survival interval of three months or less were considered to have experienced early mortality. Cancer-specific death was recorded and used in the study. In terms of American Joint Committee on Cancer and Extent of Disease classification, T and N stages were used for analysis. Race was divided into black, white, others, and unknown, the others of race included American Indian, AK Native, Asian, and Pacific Islander.

### Model training

Selection of model features was determined by subgroup analysis of clinical characteristics in the training group, and significant variables were included as the input features of model building. Five machine learning techniques, including an artificial neural network, gradient boosting decision tree, eXGBoosting machine, decision tree, and support vector machine, were investigated in the study to construct an ensemble machine learning model. Each model received the same input features. These models are widely used for binary classification issues in the field of medicine, and this study chose a wide range of models to reflect this. To further explain, gradient boosting decision tree frequently conducts well with risk classification, but an ensemble was introduced to further improve model robustness in the study. Combining the outputs of the artificial neural network, gradient boosting decision tree, eXGBoosting machine, decision tree, and support vector machine, ensemble machine learning can use models created by numerous machine learning techniques to make predictions. Particularly, ensemble models frequently produce superior predicting performance than individual machine learning models (20, 21). Broad upper and lower bounds were applied to grid and random hyperparameter searches to explore the optimal hyperparameters, and the area under the receiver operating characteristic curve (AUROC) was the primary metric to evaluate the prediction performance after the optimal hyperparameters were finally determined, helping to largely avoid underfitted and overfitted conditions.

### Model validation

The AUROC was calculated for model discrimination during model evaluation. The models’ capacity for discrimination refers to their power to discern between favorable and unfavorable outcomes. The density probability curve and discrimination slope were used in the analysis as additional indicators showing model discrimination. Brier score and visual examination of calibration plots were used to evaluate model calibration, which reflects the consistency between anticipated and observed outcomes. The predicted risk of an event developing vs. the observed risk were plotted in calibration plots, and the calibration slope and intercept-in-large were derived for each plot. For each machine learning model, a clinical net benefit was also calculated using decision curve analysis; this measure of value was accomplished by making decisions based on model predictions. For each model, other key performance measures included specificity, sensitivity, and accuracy.

### Statistical analysis

Using the *t*-test for continuous variables and chi-square test or adjusted continuity chi-square test for proportional variables, the clinical characteristics between patients in the training and testing groups were compared. In order to interpret feature contributions, in terms of the ensemble machine learning model, Shaley Additive Explanation (SHAP) was utilized. Patients were categorized into two risk groups using the ensemble machine learning model, stratified by the ideal cut-off value (threshold). The chi-square test was used to compare the difference of the actual probability of developing early mortality among patients in the high- and low-risk groups. The Kaplan–Meier method and log-rank test were conducted to create the survival curve among patients stratified by risk groups. The statistical tools used for these analyses included the R statistical software (R Project for Statistical Computing, version 4.1.2) and Python (version 3.9.7). Statistical significance was defined as a two-sided p-value of 0.05.

## Results

### Process of screening and clinicopathology

**Figure 1:**
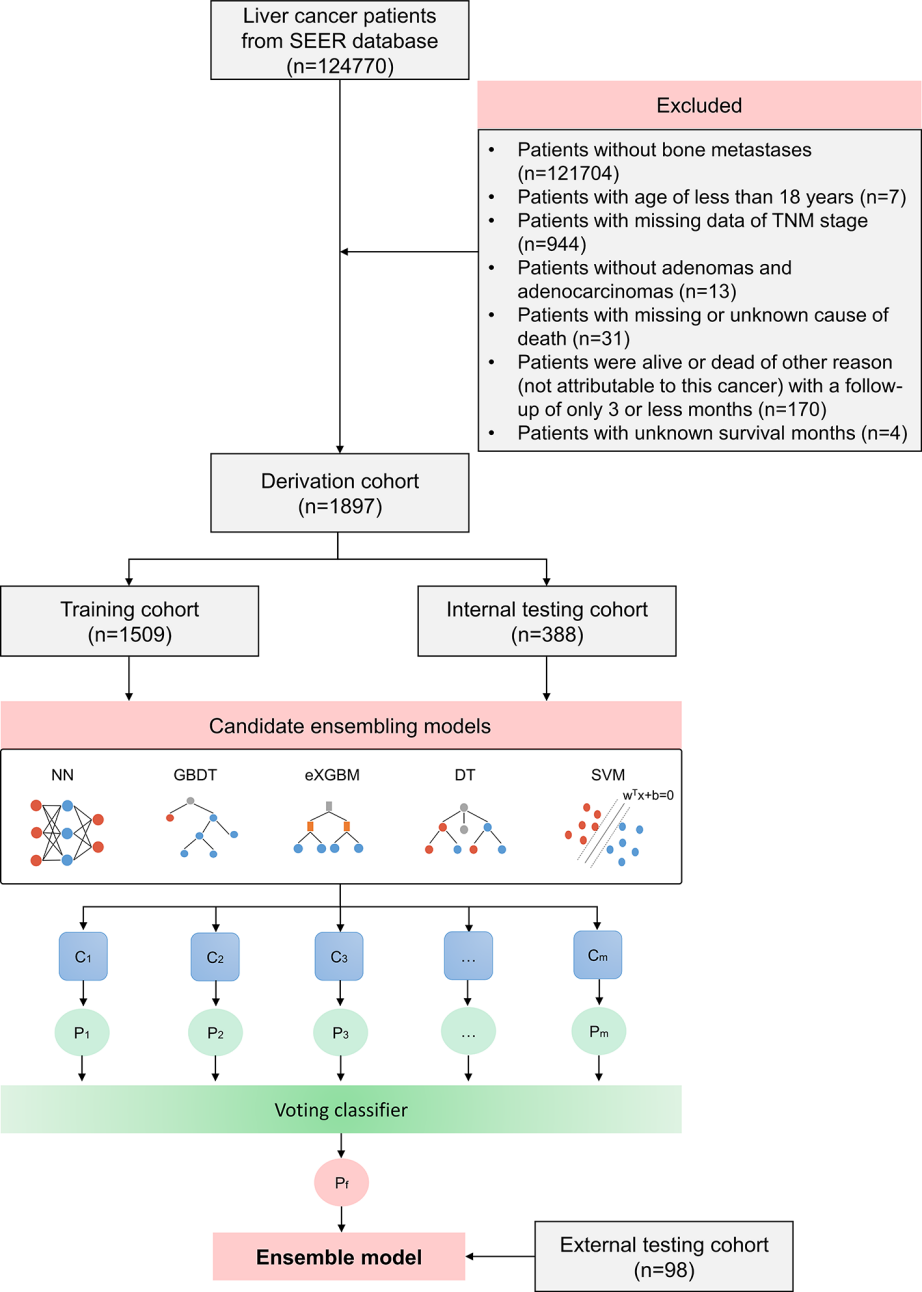
Flow chart outlining patient’s enrollment, study design, and ensemble machine learning technique.

The study included 124,770 people with liver cancer in total. A cohort of 1,897 individuals from the SEER database who had been histologically determined to have HCC with bone metastases were included based on the screening criteria (**Figure 1**). The baseline clinical characteristics of patients are shown in **Table 1**. The average age of the patients was 65.04 (10.20) years, with the majority of them being men (85.6%), Caucasian (72.6%), and married (46.4%). A large number of tumors were T3 (29.7%) and N0 (62.3%) disease. Up to 62.2% of patients had positive AFP results. In addition to bone metastases, brain metastases, liver metastases, and lung metastases accounted for 3.2%, 7.2%, and 23.0%, respectively, indicating relatively heavy metastatic illness. Only 2.6% of patients received cancer-specific surgery, while 0.6% of patients underwent lymph node surgery. In the entire cohort of patients, 39.7% patients received radiation and 38.7% patients had chemotherapy. There were 55.5% of patients who had events (early mortality from HCC). The median survival time was 3.0 months (range: 0.0–98.0 months).

**Table 1:** Baseline clinical characteristics of the entire cohort.

Characteristics	Overall
n	1897
Age (mean (SD))	65.04 (10.20)
Sex	
Female	274 (14.4)
Male	1623 (85.6)
Race (%)	
Black	279 (14.7)
Others	234 (12.3)
Unknown	7 (0.4)
White	1377 (72.6)
Marital status (%)	
Married	881 (46.4)
Others	474 (25.0)
Single	443 (23.4)
Unknown	99 (5.2)
T stage (%)	
T0	20 (1.1)
T1	435 (22.9)
T2	241 (12.7)
T3	564 (29.7)
T4	171 (9.0)
TX	466 (24.6)
N stage (%)	
N0	1181 (62.3)
N1	362 (19.1)
NX	354 (18.7)
Fibrosis score (%)	
Ishak 0–4	59 (3.1)
Ishak 5–6	254 (13.4)
Unknown	1584 (83.5)
AFP level (%)	
Negative	239 (12.6)
Positive	1179 (62.2)
Unknown	479 (25.3)
Tumor size (mm, %)	
Less than 45	105 (5.5)
46–85	143 (7.5)
More than 86	253 (13.3)
Unknown	1396 (73.6)
Brain metastases (%)	
No	1764 (93.0)
Unknown	72 (3.8)
Yes	61 (3.2)
Liver metastases (%)	
No	1690 (89.1)
Unknown	70 (3.7)
Yes	137 (7.2)
Lung metastases (%)	
No	1388 (73.2)
Unknown	72 (3.8)
Yes	437 (23.0)
Surgery of lymph (%)	
Yes	12 (0.6)
None/unknown	1885 (99.4)
Cancer-directed surgery (%)	
Yes	49 (2.6)
None/unknown	1848 (97.4)
Radiation (%)	
Yes	753 (39.7)
None/unknown	1144 (60.3)
Chemotherapy (%)	
Yes	734 (38.7)
None/unknown	1163 (61.3)
Early death (%)	
Yes	1052 (55.5)
No	845 (44.5)

SD, Standard deviation; T, tumor; N, node; AFP, alpha fetoprotein.

### Development of the ensemble model

A comparison of clinical characteristics was operated between patients in the training and internal testing cohort, and it demonstrated that the two cohorts were comparable because no significant difference was found in the distribution of the clinical characteristics (**Table 2**). In the training cohort, the study found that early mortality patients in the training cohort were more likely to be men (p = 0.019), single (p = 0.004), with advanced T (p = 0.025) and N (p = 0.001) stage, unknown fibrosis score (p = 0.040), positive AFP level (p = 0.032), larger tumor size (p = 0.001), lung metastases (p < 0.001), less cancer-directed surgery (p < 0.001), less radiation (p < 0.001), and less chemotherapy (p < 0.001), whereas other clinical characteristics were insignificant (**Table 3**). Thus, in order to train and improve the models, the aforementioned 11 clinical criteria were used, and the best hyperparameters were found after grid and random hyperparameter searches for each model (**Table 4**). At last, the ensemble machine learning model was developed in a soft-voting method to combine the results from the five machine learning algorithms in the study, including the artificial neural network, gradient boosting decision tree, eXGBoosting machine, decision tree, and support vector machine.

**Table 2:** Clinical characteristics among patients stratified by the splitting group.

Characteristics	Training cohort	Internal testing cohort	p
n	1509	388	
Age [mean (SD)]	64.98 (10.23)	65.28 (10.07)	0.608
Sex (%)			0.681
Female	221 (14.6)	53 (13.7)	
Male	1288 (85.4)	335 (86.3)	
Race (%)			0.978
Black	222 (14.7)	57 (14.7)	
Others	185 (12.3)	49 (12.6)	
Unknown	6 (0.4)	1 (0.3)	
White	1096 (72.6)	281 (72.4)	
Marital status (%)			0.399
Married	694 (46.0)	187 (48.2)	
Others	390 (25.8)	84 (21.6)	
Single	348 (23.1)	95 (24.5)	
Unknown	77 (5.1)	22 (5.7)	
T stage (%)			0.821
T0	15 (1.0)	5 (1.3)	
T1	343 (22.7)	92 (23.7)	
T2	187 (12.4)	54 (13.9)	
T3	456 (30.2)	108 (27.8)	
T4	140 (9.3)	31 (8.0)	
TX	368 (24.4)	98 (25.3)	
N stage (%)			0.435
N0	948 (62.8)	233 (60.1)	
N1	288 (19.1)	74 (19.1)	
NX	273 (18.1)	81 (20.9)	
Fibrosis score (%)			0.184
Ishak 0–4	45 (3.0)	14 (3.6)	
Ishak 5–6	192 (12.7)	62 (16.0)	
Unknown	1272 (84.3)	312 (80.4)	
AFP level (%)			0.353
Negative	189 (12.5)	50 (12.9)	
Positive	928 (61.5)	251 (64.7)	
Unknown	392 (26.0)	87 (22.4)	
Tumor size (mm, %)			0.063
Less than 45	92 (6.1)	13 (3.4)	
46–85	114 (7.6)	29 (7.5)	
More than 86	190 (12.6)	63 (16.2)	
Unknown	1113 (73.8)	283 (72.9)	
Brain metastases (%)			0.707
No	1400 (92.8)	364 (93.8)	
Unknown	60 (4.0)	12 (3.1)	
Yes	49 (3.2)	12 (3.1)	
Liver metastases (%)			0.563
No	1343 (89.0)	347 (89.4)	
Unknown	59 (3.9)	11 (2.8)	
Yes	107 (7.1)	30 (7.7)	
Lung metastases (%)			0.797
No	1106 (73.3)	282 (72.7)	
Unknown	59 (3.9)	13 (3.4)	
Yes	344 (22.8)	93 (24.0)	
Surgery of lymph (%)			1.000
Yes	10 (0.7)	2 (0.5)	
None/unknown	1499 (99.3)	386 (99.5)	
Cancer-directed surgery (%)			0.206
Yes	43 (2.8)	6 (1.5)	
None/unknown	1466 (97.2)	382 (98.5)	
Radiation (%)			0.863
Yes	597 (39.6)	156 (40.2)	
None/unknown	912 (60.4)	232 (59.8)	
Chemotherapy (%)			0.873
Yes	582 (38.6)	152 (39.2)	
None/unknown	927 (61.4)	236 (60.8)	
Early death (%)			0.516
Yes	843 (55.9)	209 (53.9)	
No	666 (44.1)	179 (46.1)	

SD, Standard deviation; T, tumor; N, node; AFP, alpha fetoprotein.

**Table 3:** Clinical characteristics among patients stratified by early death in the training cohort.

Characteristics	Overall	Early death		p
		No	Yes	
n	1509	666	843	
Age [mean (SD)]	64.98 (10.23)	65.07 (10.08)	64.92 (10.35)	0.779
Sex (%)				0.019
Female	221 (14.6)	81 (12.2)	140 (16.6)	
Male	1288 (85.4)	585 (87.8)	703 (83.4)	
Race (%)				0.668
Black	222 (14.7)	98 (14.7)	124 (14.7)	
Others	185 (12.3)	74 (11.1)	111 (13.2)	
Unknown	6 (0.4)	3 (0.5)	3 (0.4)	
White	1096 (72.6)	491 (73.7)	605 (71.8)	
Marital status (%)				0.004
Married	694 (46.0)	340 (51.1)	354 (42.0)	
Others	390 (25.8)	164 (24.6)	226 (26.8)	
Single	348 (23.1)	133 (20.0)	215 (25.5)	
Unknown	77 (5.1)	29 (4.4)	48 (5.7)	
T stage (%)				0.025
T0	15 (1.0)	5 (0.8)	10 (1.2)	
T1	343 (22.7)	175 (26.3)	168 (19.9)	
T2	187 (12.4)	82 (12.3)	105 (12.5)	
T3	456 (30.2)	201 (30.2)	255 (30.2)	
T4	140 (9.3)	49 (7.4)	91 (10.8)	
TX	368 (24.4)	154 (23.1)	214 (25.4)	
N stage (%)				0.001
N0	948 (62.8)	451 (67.7)	497 (59.0)	
N1	288 (19.1)	103 (15.5)	185 (21.9)	
NX	273 (18.1)	112 (16.8)	161 (19.1)	
Fibrosis score (%)				0.040
Ishak 0–4	45 (3.0)	28 (4.2)	17 (2.0)	
Ishak 5–6	192 (12.7)	87 (13.1)	105 (12.5)	
Unknown	1272 (84.3)	551 (82.7)	721 (85.5)	
AFP level (%)				0.032
Negative	189 (12.5)	100 (15.0)	89 (10.6)	
Positive	928 (61.5)	395 (59.3)	533 (63.2)	
Unknown	392 (26.0)	171 (25.7)	221 (26.2)	
Tumor size (mm, %)				0.001
Less than 45	92 (6.1)	50 (7.5)	42 (5.0)	
46–85	114 (7.6)	65 (9.8)	49 (5.8)	
More than 86	190 (12.6)	69 (10.4)	121 (14.4)	
Unknown	1113 (73.8)	482 (72.4)	631 (74.9)	
Brain metastases (%)				0.519
No	1400 (92.8)	623 (93.5)	777 (92.2)	
Unknown	60 (4.0)	25 (3.8)	35 (4.2)	
Yes	49 (3.2)	18 (2.7)	31 (3.7)	
Liver metastases (%)				0.071
No	1343 (89.0)	602 (90.4)	741 (87.9)	
Unknown	59 (3.9)	28 (4.2)	31 (3.7)	
Yes	107 (7.1)	36 (5.4)	71 (8.4)	
Lung metastases (%)				<0.001
No	1106 (73.3)	556 (83.5)	550 (65.2)	
Unknown	59 (3.9)	24 (3.6)	35 (4.2)	
Yes	344 (22.8)	86 (12.9)	258 (30.6)	
Surgery of lymph (%)				0.182
Yes	10 (0.7)	7 (1.1)	3 (0.4)	
None/unknown	1499 (99.3)	659 (98.9)	840 (99.6)	
Cancer-directed surgery (%)				<0.001
Yes	43 (2.8)	36 (5.4)	7 (0.8)	
None/unknown	1466 (97.2)	630 (94.6)	836 (99.2)	
Radiation (%)				<0.001
Yes	597 (39.6)	376 (56.5)	221 (26.2)	
None/unknown	912 (60.4)	290 (43.5)	622 (73.8)	
Chemotherapy (%)				<0.001
Yes	582 (38.6)	409 (61.4)	173 (20.5)	
None/unknown	927 (61.4)	257 (38.6)	670 (79.5)	

SD, Standard deviation; T, tumor; N, node; AFP, alpha fetoprotein.

**Table 4:** Models and their hyperparameters.

Models	Hyperparameters
Neural Network	MLPClassifier (alpha=1e-05, hidden_layer_sizes=100, random_state=42)
Gradient Boosting Decision Tree	GradientBoostingClassifier (max_depth=1, max_features=‘auto’, min_samples_leaf=186, min_samples_split=179, n_estimators=102, random_state=42)
eXGBoosting Machine	XGBClassifier (base_score=0.5, booster=‘gbtree’, colsample_bylevel=1, colsample_bynode=1, colsample_bytree=1, enable_categorical=False, gamma=0, gpu_id=-1, importance_type=None, interaction_constraints=‘‘, learning_rate=0.125, max_delta_step=0, max_depth=75, min_child_weight=56, missing=nan, monotone_constraints=‘()’, n_estimators=36, n_jobs=8, num_parallel_tree=1, predictor=‘auto’, random_state=42, reg_alpha=0, reg_lambda=1, scale_pos_weight=1, subsample=1, tree_method=‘exact’, use_label_encoder=False, validate_parameters=1, verbosity=None)
Decision Tree	DecisionTreeClassifier (max_depth=24, max_features=‘auto’, min_samples_leaf=100, min_samples_split=173, random_state=42)
Support Vector Machine	SVC (C=0.09837555188414593, gamma=0.11638567021515211, probability=True)

### Validation of the ensemble model

**Figure 2:**
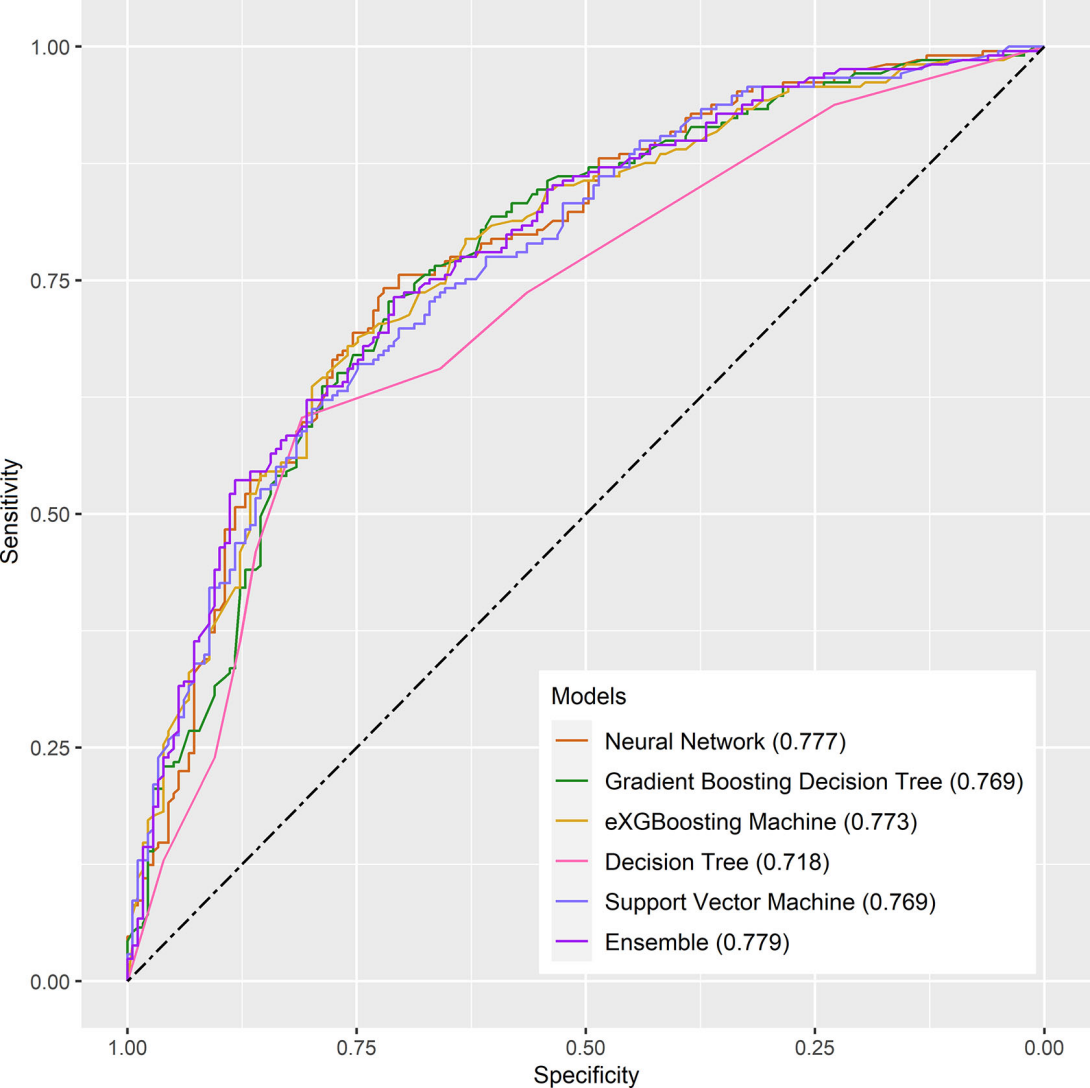
The receiver operating characteristic curves for the machine learning models in the internal testing cohort.

**Figure 3:**
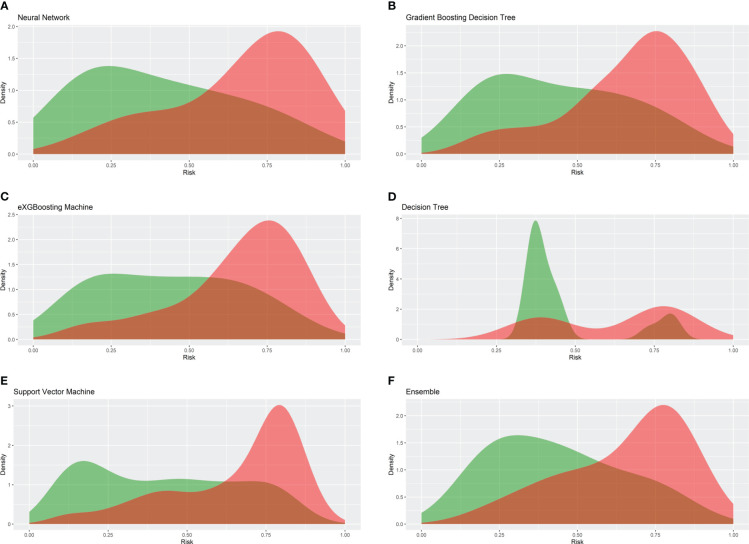
Density cures of the machine learning models in the internal testing cohort. **(A)** Neural network; **(B)** gradient boosting decision tree; **(C)** eXGBoosting machine; **(D)** decision tree; **(E)** support vector machine; **(F)** ensemble model.

**Figure 4:**
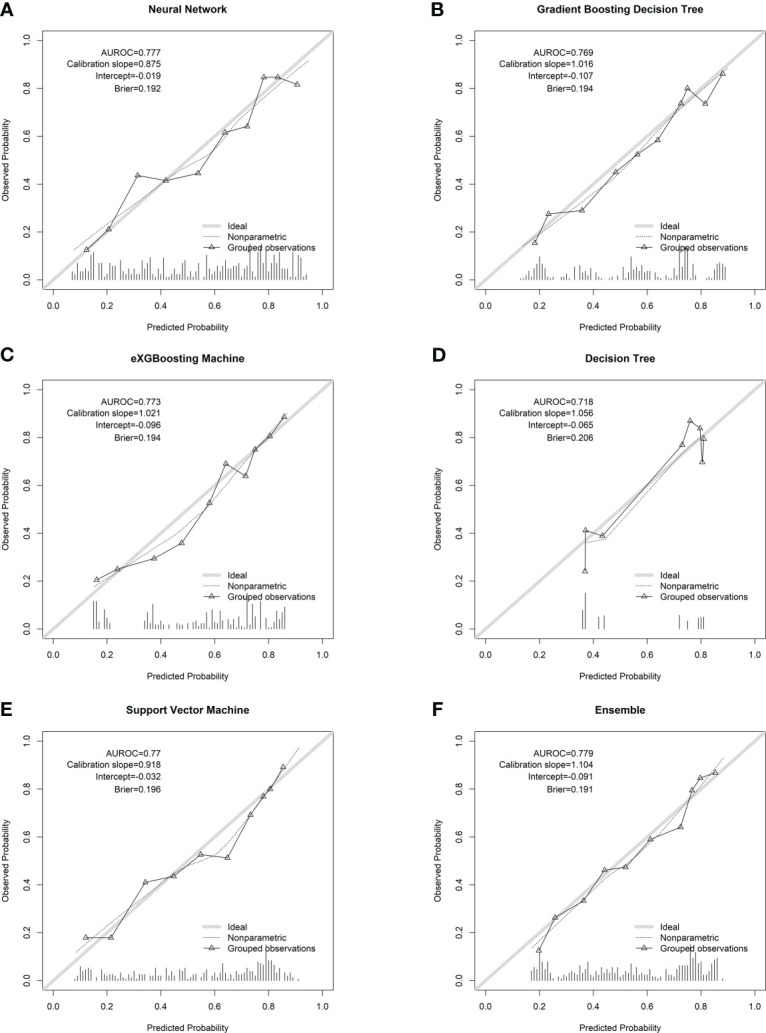
Calibration plots of the machine learning models in the internal testing cohort. **(A)** Neural network; **(B)** gradient boosting decision tree; **(C)** eXGBoosting machine; **(D)** decision tree; **(E)** support vector machine; **(F)** ensemble model.

**Figure 5:**
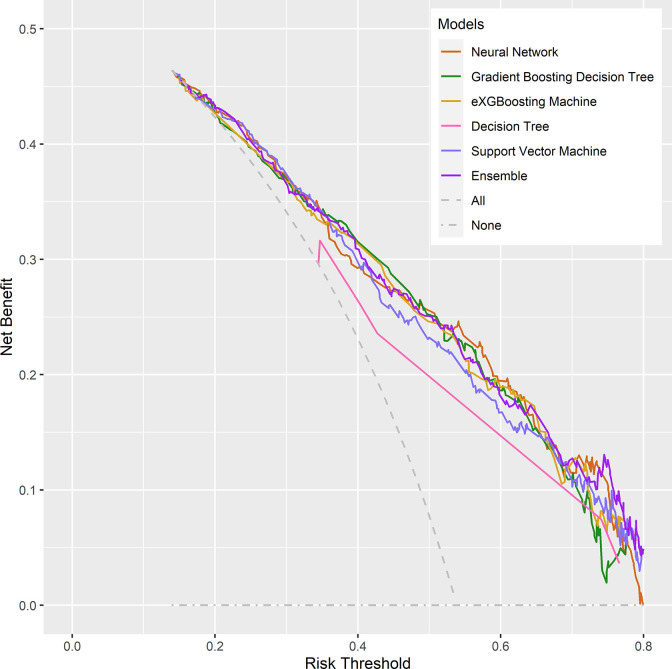
Decision curve analysis of the machine learning models in the internal testing cohort.

**Figure 6:**
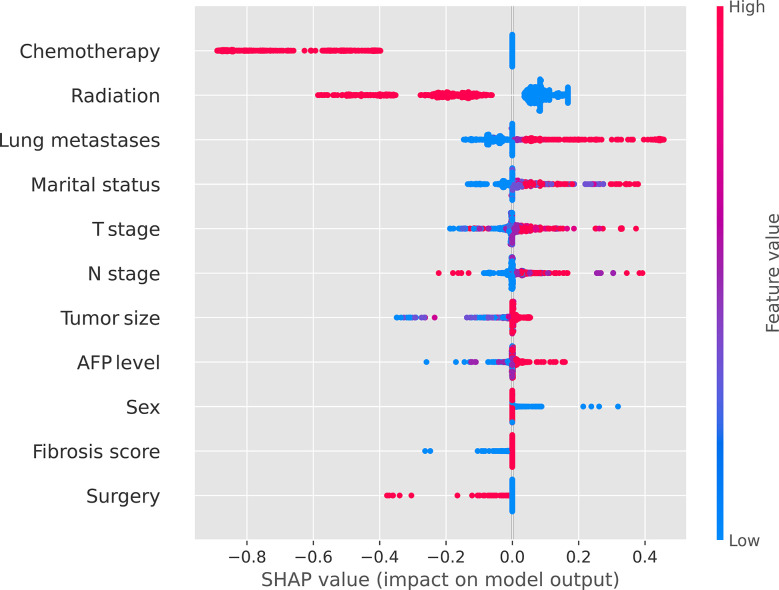
Feature importance in terms of the ensemble machine learning model.

Internal validation of the model was operated in the internal testing cohort, and external validation was performed in the external testing cohort. The baseline characteristics of the external testing cohort are shown in **Supplementary Table 1**. Application of the ensemble model in the internal testing population yielded an AUROC of 0.779 (95% CI: 0.727–0.820) (**Figure 2**), which was the largest AUROC among all models, suggesting optimal discrimination in the study. The neural network model had the second-highest AUROC, which was 0.777 (95% CI: 0.730–0.823), and was followed by the eXGBoosting machine model. The external validation showed the AUROC of the ensemble model was 0.764 (95% CI: 0.642–0.886) (**Supplementary Figure 1**). Each model’s probability density curve is shown in **Figure 3**, which reveals that most models exhibited favorable discrimination with a sizable portion of separation. The similar trend of density curve was also observed in the external validation according to the ensemble model (**Supplementary Figure 2**). The majority of models displayed positive discrimination, as shown by the calculation of the discrimination slope, which was defined as the mean difference between actual and observed risk probabilities of occurrences (**Supplementary Figure 3**). External validation elucidated that the discrimination slope was also up to 0.211 in the ensemble model (**Supplementary Figure 4**). Of note, other machine learning models produced a higher Brier score than the ensemble machine learning model, indicating a bigger prediction error. **Table 5** summarizes additional indicators in greater detail. Calibration plots are displayed in **Figures 4**, **5** shows the decision curve for each model in the study, showing that models, in particular the ensemble machine learning model, had good clinical usefulness. The calibration plot of the ensemble model in the external validation is shown in **Supplementary Figure 5**. It showed the calibration curve was not close to the ideal reference line, although the calibration slope was near to 1. To further improve the calibration of the ensemble model, we revised the model *via* subtracting 20.0% in each predicted risk of early mortality. Thus, the new revised calibration plot was provided (**Supplementary Figure 6**), and it demonstrated that the calibration of the model was further improved. In addition, the AUROC, Baier score, and calibration slope were all improved after the revision of model (**Table 5**). Based on the above findings, although the decision tree had the poorest prediction performance based on the AUROC, it still had advantages based on the intercept-in-large (-0.065) and specificity (0.810). The intercept-in-large was very near to 0, and the specificity was the highest, among all machine learning models. Thus, the decision tree model was also included to develop the ensemble machine learning model. The study found that the top three important features included chemotherapy, radiation, and lung metastases (**Figure 6**), according to feature importance analysis using the ensemble machine learning model.

**Table 5:** Key performance indicators of models.

Models	AUROC	Baier score	Intercept-in-large	Calibration slope	Specificity	Sensitivity	Accuracy	Threshold
Neural Network	0.777 (0.730–0.823)	0.192	-0.019	0.875	0.721	0.742	0.732	0.571
Gradient Boosting Decision Tree	0.769 (0.722–0.817)	0.194	-0.107	1.016	0.715	0.727	0.722	0.578
eXGBoosting Machine	0.773 (0.727–0.820)	0.194	-0.096	1.021	0.760	0.679	0.716	0.624
Decision Tree	0.718 (0.668–0.769)	0.206	-0.065	1.056	0.810	0.603	0.698	0.587
Support Vector Machine	0.769 (0.723–0.816)	0.196	0.918	-0.032	0.799	0.612	0.698	0.669
Ensemble^a^	0.779 (0.733–0.825)	0.191	-0.091	1.104	0.709	0.732	0.722	0.541
Ensemble^b^	0.764 (0.642–0.886)	0.195	-1.022	1.083	0.778	0.692	0.755	0.549
Ensemble^c^	0.778 (0.658–0.887)	0.159	-0.064	0.999	0.778	0.692	0.755	0.349

AUROC, Area under the receiver operating characteristic curve.

a indicates internal testing cohort;

b indicates external testing cohort;

c indicates external testing cohort after model revision.

### Risk category

Reclassification of patients was conducted using the ensemble machine learning model’s threshold of 54.1%. The low-risk group included patients with a forecasted risk probability of 54.1% or less, whereas the high-risk group included patients with a predicted risk probability of more than 54.1%. The actual probability of early mortality was significantly different between the two risk groups (p < 0.001, **Table 6**). The Kaplan–Meier survival curve also showed that patients in the high-risk group had significant shorter survival time in comparison to patients in the low-risk group (p < 0.001, log-rank test, **Supplementary Figure 7**).

**Table 6:** Risk stratification of patients in the internal validation cohort based on the ensemble model.

Risk group	Patients (n = 388)	Predicted risk	Observed risk	p
Low risk (≤0.541)	185	34.52%	31.35%	<0.001
High risk (>0.541)	203	74.11%	74.38%	

## Discussion

This study constructed a model to predict early mortality among HCC patients with bone metastases, and the model was developed using the ensemble machine learning technique that combined the results of multiple machine-learning algorithms, including an artificial neural network, gradient boosting decision tree, eXGBoosting machine, decision tree, and support vector machine. The ensemble model outperformed other algorithms in terms of both discrimination and calibration, as evidenced by its greatest AUROC and lowest Brier score. This model might be a helpful predictive tool to determine the likelihood that these individuals would develop early death and to aid in therapeutic decision-making.

In HCC patients with bone metastases, the early mortality rate was 55.5%, showing a comparatively high rate of early death in these patients. According to current literature, the median survival period was only about 2.8 to 3.3 months among HCC patients with bone metastases (12–14). In the present study, the median survival time was 3.0 months (range: 0.0–98.0 months), and this number was consistent with other studies (12–14). But a retrospective study which was conducted by Hirai et al. (8) reported that the median survival was up to 11.07 months after the diagnosis of bone metastases among HCC patients. In addition, a study with small sample size found that the median survival time was 10.0 months among patients with skeletal metastases due to HCC after surgical treatment (16). After analyzing 37 HCC patients with bone metastases, Kim et al. showed that the median survival was 6.2 months (18). The incidence of early death was 26.5% in the external testing cohort, and this number was significantly lower than that in the cohort from the SEER database. The difference might be that the external testing cohort had a significantly higher rate of cancer surgery (43.9% vs. 2.6%) and chemotherapy (67.3% vs. 38.7%), as compared to the patients from the SEER cohort. In addition, HCC patients with bone metastases from the SEER database were initially diagnosed, whereas in the external testing cohort HCC patients who later developed bone metastases after initial HCC diagnosis were enrolled for analysis. The aforesaid discrepancy may be explained by the small size of the study sample and the population variability.

Numerous researches have looked into the potential risk and protective factors for determining the likelihood that HCC patients with bone metastases would survive. For instance, Guo et al. (14) revealed that married status was independently associated with better survival outcome among HCC patients with bone metastases at initial diagnosis after analyzing 1567 cases from the SEER database. Japanese researchers showed that age of more than 75 years, hepatitis C-virus etiology, and Child-Pugh class B/C were significantly relevant to a worse survival outcome after enrolling 76 patients, and the study also pointed out that pathological fracture or paralysis had no impact on the survival (8). In addition, Honda et al. (15) also demonstrated that Child-Pugh grade and T stage were correlated with overall survival among 99 HCC patients with bone metastases. In a retrospective study of 42 cases, the number of bone metastases and Child-Pugh class were found as independent prognostic factors. However, In a retrospective study of 37 HCC patients presenting with bone metastases, it showed that the presence of ascites was the sole risk factor for survival, while other variables, such as age, gender, performance status, Child-Pugh class, AFP, and treatment for HCC were insignificant (18). Regarding therapeutic approaches, primary tumor surgery (14), chemotherapy (12), radiation (17), and palliation care (17) were proved to be beneficial for survival outcome among those patients. In the present study, feature importance demonstrated that the top three most important features were chemotherapy, radiation, and lung metastases, and the impact of the three clinical characteristics on survival has been confirmed in previous studies (22). Chemotherapy and radiation were protective factors for early death. In addition, among HCC patients, lung metastases showed a worse prognosis than bone metastases (6), demonstrating that lung metastases had a significant negative impact on survival.

For patients with HCC, a number of survival prediction models have been put forth to forecast the outcome of survival. For example, Liang et al. (23) used the Cancer Genome Atlas cohort to construct a survival prediction model for HCC patients utilizing 10 ferroptosis-related genes, and the International Cancer Genome Consortium cohort to validate the model. The AUROC for estimating 1-year survival was 0.68, 2-year survival was 0.69, and 3-year survival was 0.72. Yan et al. (24) established a survival prediction model after analyzing 3620 patients with early HCC and the model consisted of eight variables including age, race, grade, T stage, surgery, chemotherapy, tumor size, and marital status. The 3- and 5-year AUROC were 0.767 and 0.766, respectively. More recently, after enrolling 2514 HCC patients in a multicenter database, a nomogram prediction model for survival was proposed using eight clinical characteristics for patients with and without adjuvant transcatheter arterial chemoembolization, and validation of the nomogram showed that the C-index was slightly above 0.75 (25). Liu et al. (26) developed a radiomics nomogram to predict the overall survival of HCC patients after hepatectomy. To begin with, this study constructed a radiomics signature in terms of seven overall survival related texture parameters, and then the radiomics signature incorporating with other four clinical characteristics (AFP, platelet-to-lymphocyte ratio, tumor size, and microvascular invasion) was used to develop the radiomics nomogram. The radiomics nomogram had an AUROC value of 0.747 in the training cohort and 0.777 in the validation cohort. However, studies on developing survival prediction specifically among HCC patients with bone metastases were scarce. To our knowledge, this study was the first to construct an accurate model to predict early mortality specifically among HCC patients with bone metastases using the ensemble machine learning technique, and this technique was able to combine the results of multiple machine-learning algorithms. Of note, the ensemble model had favorable discrimination and calibration in terms of AUROC (0.779) and Brier score (0.191), respectively. Notably, as compared to the AUROC in the above studies, our study had the highest AUROC, suggesting the accuracy of the prediction model was favorable.

Reclassification of patients showed that actual probability of early mortality was significant difference between the two risk groups (74.38% vs. 31.35%, p < 0.001). To be specific, patients in the high-risk group were 2.37 times more likely to suffer early death as compared to patients in the low-risk group. The Kaplan–Meier survival curve also demonstrated that patients in the high-risk group had significant shorter survival time in comparison to patients in the low-risk group. Patients in the high-risk group may therefore require greater care. Surgery may not be advised for those individuals because they were at a high danger of passing away within 3 months, would not have enough time to recuperate from surgery, and had slim prospects of ever benefiting from it. In addition, a multidisciplinary cooperation was recommended to manage HCC patients with bone metastases due to its complexity (11), and if there were no specifically targeted drugs, the therapeutic aim of treatments is directed at palliation of symptoms (11).

### Limitations

The restrictions of this study are outlined below: (1) Because some clinical criteria, such as Child-Pugh grade, are not available in the SEER database, this study’s selection of variables is constrained. (2) The information that was taken from the SEER database was on the condition at the time of the initial diagnosis, suggesting that bone metastases that occur in the later stages may not have been documented. (3) The model showed positive predictive performance in both the internal and external validation, but additional external validation is still needed to increase the model’s generalizability.

## Conclusions

In conclusion, the ensemble machine learning model shows promising prediction performance for early mortality among HCC patients with bone metastases. This model can be a prognostic tool to predict the survival outcome of those patients and facilitate clinical decision-making. Surgery might not be advised for patients in the high-risk group because they had a high chance of passing away within 3 months. For a subset of patients, chemotherapy, radiation therapy, and the avoidance or treatment of lung metastases are advised due to their positive effects on survival.

## Data availability statement 

Publicly available datasets were analyzed in this study. Training and internal testing data are available at https://seer.cancer.gov/. External testing data are available under reasonable request to the corresponding authors.

## Ethics statement 

This study was approved by Hainan Hospital of Chinese PLA General Hospital and patients gave informed written consent prior to data collection. Written informed consent for participation was not required for this study in accordance with the national legislation and the institutional requirements.

## Author contributions

All authors conceived and designed the analysis; ZL, SW, XC, and QY oversaw data collection, YQ and ML performed the analysis, and all authors provided clinical interpretation of the findings. MY and ML drafted the manuscript. The corresponding author has full access to all the data in the study and had final responsibility for the decision to submit for publication. All authors contributed to the article and approved the submitted version.

## Supplementary material

Supplementary Figure 1 The receiver operating characteristic curve for the ensemble model in the external testing cohort.

Supplementary Figure 2 Density cure for the ensemble model in the external testing cohort.

Supplementary Figure 3 Discrimination slope of the models in the internal testing cohort. A, Neural network; B, gradient boosting decision tree; C, eXGBoosting machine; D, decision tree; E, support vector machine; F, ensemble model.

Supplementary Figure 4 Discrimination slope of the ensemble model in the external testing cohort.

Supplementary Figure 5 Calibration plot of the ensemble model in the external testing cohort.

Supplementary Figure 6 Calibration plot of the ensemble model in the external testing cohort after model revision.

Supplementary Figure 7 Kaplan–Meier survival curve among patients stratified by risk group (p < 0.0001, log-rank test).
