# Supplementary material for: Development and validation of an ensemble machine-learning model for predicting early mortality among patients with bone metastases of hepatocellular carcinoma
Source: Front Oncol. 2023 Feb 20;13:1144039. doi: 10.3389/fonc.2023.1144039 (PMC9986604; doi:10.3389/fonc.2023.1144039)
Supplement: Supplementary file 1 [file Table_1.docx]

| **Supplementary table 1:** Baseline characteristics of patients in the external testing cohort. | |
| --- | --- |
| **Characteristics** | **Overall** |
| n | 98 |
| Sex (female/male, %) | 11/87 (11.2/88.8) |
| Marital status (married/single, %) | 90/8 (91.8/8.2) |
| T stage (%) |  |
| T1 | 4 (4.1) |
| T2 | 20 (20.4) |
| T3 | 30 (30.6) |
| T4 | 36 (36.7) |
| TX | 8 (8.2) |
| N stage (%) |  |
| N0 | 18 (18.4) |
| N1 | 36 (36.7) |
| NX | 44 (44.9) |
| Fibrosis score (%) |  |
| Ishak 0-4 | 29 (29.6) |
| Ishak 5-6 | 57 (58.2) |
| Unknown | 12 (12.2) |
| AFP level (%) |  |
| Negative | 28 (28.6) |
| Unknown | 8 (8.2) |
| Positive | 62 (63.3) |
| Tumor size (%) |  |
| Less than 45 | 36 (36.7) |
| 46-85 | 31 (31.6) |
| More than 86 | 23 (23.5) |
| Unknown | 8 (8.2) |
| Lung metastases (%) |  |
| No | 55 (56.1) |
| Unknown | 2 (2.0) |
| Yes | 41 (41.8) |
| Surgery (%) |  |
| Yes | 43 (43.9) |
| None/Unknown | 55 (56.1) |
| Radiation (%) |  |
| Yes | 34 (34.7) |
| None/Unknown | 64 (65.3) |
| Chemotherapy (%) |  |
| Yes | 66 (67.3) |
| None/Unknown | 32 (32.7) |
| Early death (%) |  |
| Yes | 26 (26.5) |
| No | 72 (73.5) |
| T, Tumor; N, Node; AFP, Alpha fetoprotein. | |
