# Supplementary figures and images for: Development and validation of an ensemble machine-learning model for predicting early mortality among patients with bone metastases of hepatocellular carcinoma
Source: Front Oncol. 2023 Feb 20;13:1144039. doi: 10.3389/fonc.2023.1144039 (PMC9986604; doi:10.3389/fonc.2023.1144039)

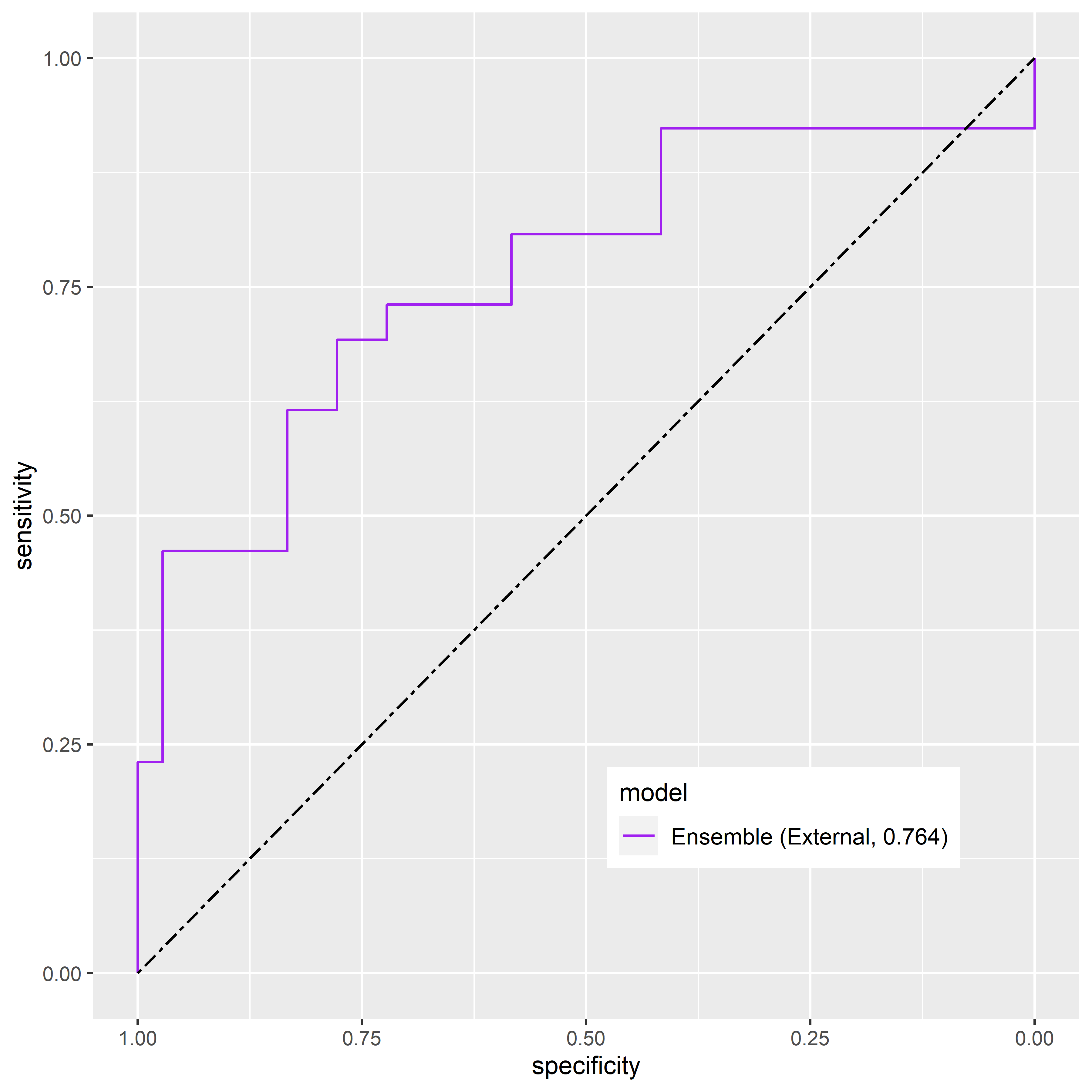

Supplement: Supplementary Figure 1 — The receiver operating characteristic curve for the ensemble model in the external testing cohort. [file Image_1.tif]

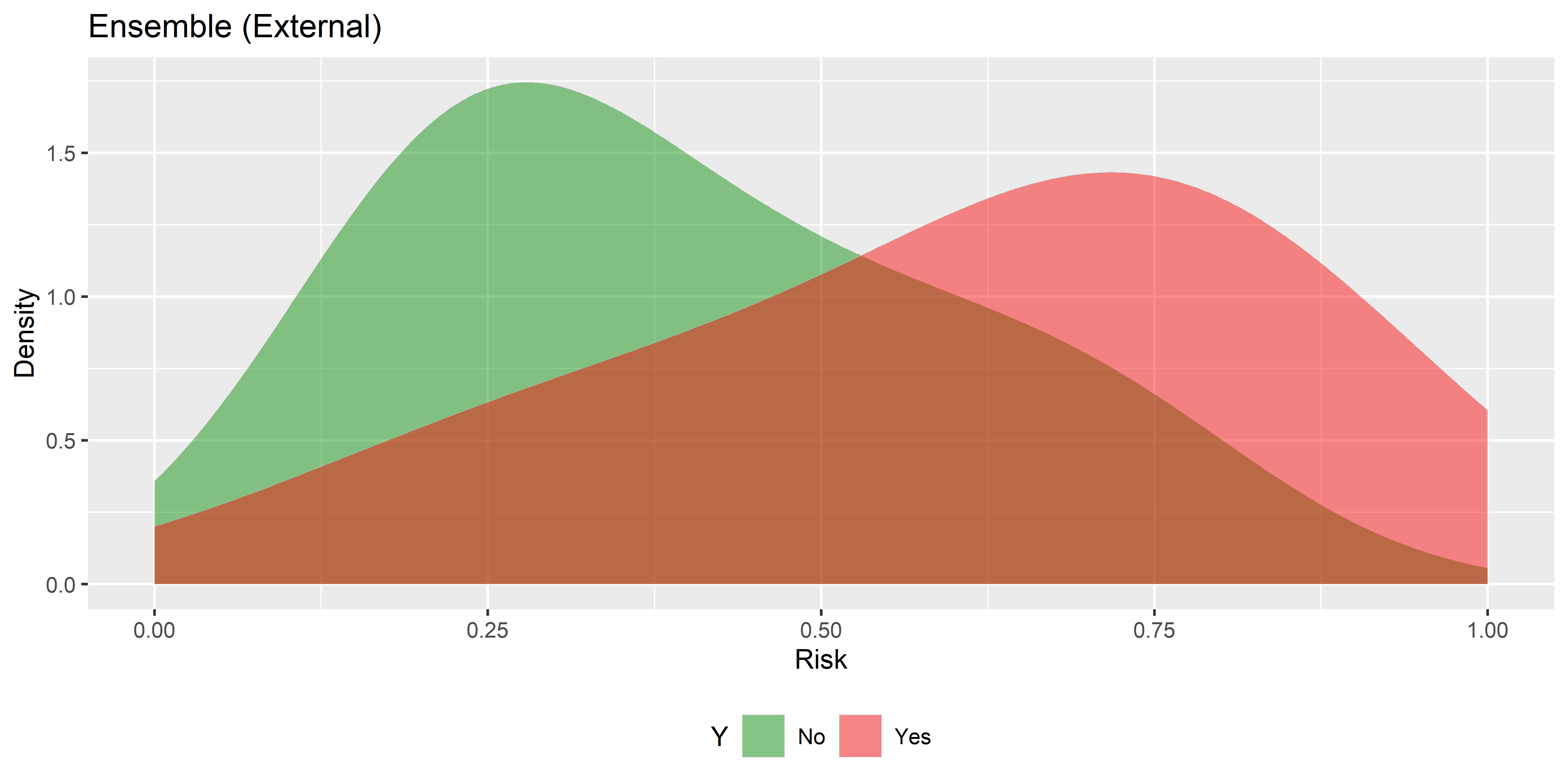

Supplement: Supplementary Figure 2 — Density cure for the ensemble model in the external testing cohort. [file Image_2.tif]

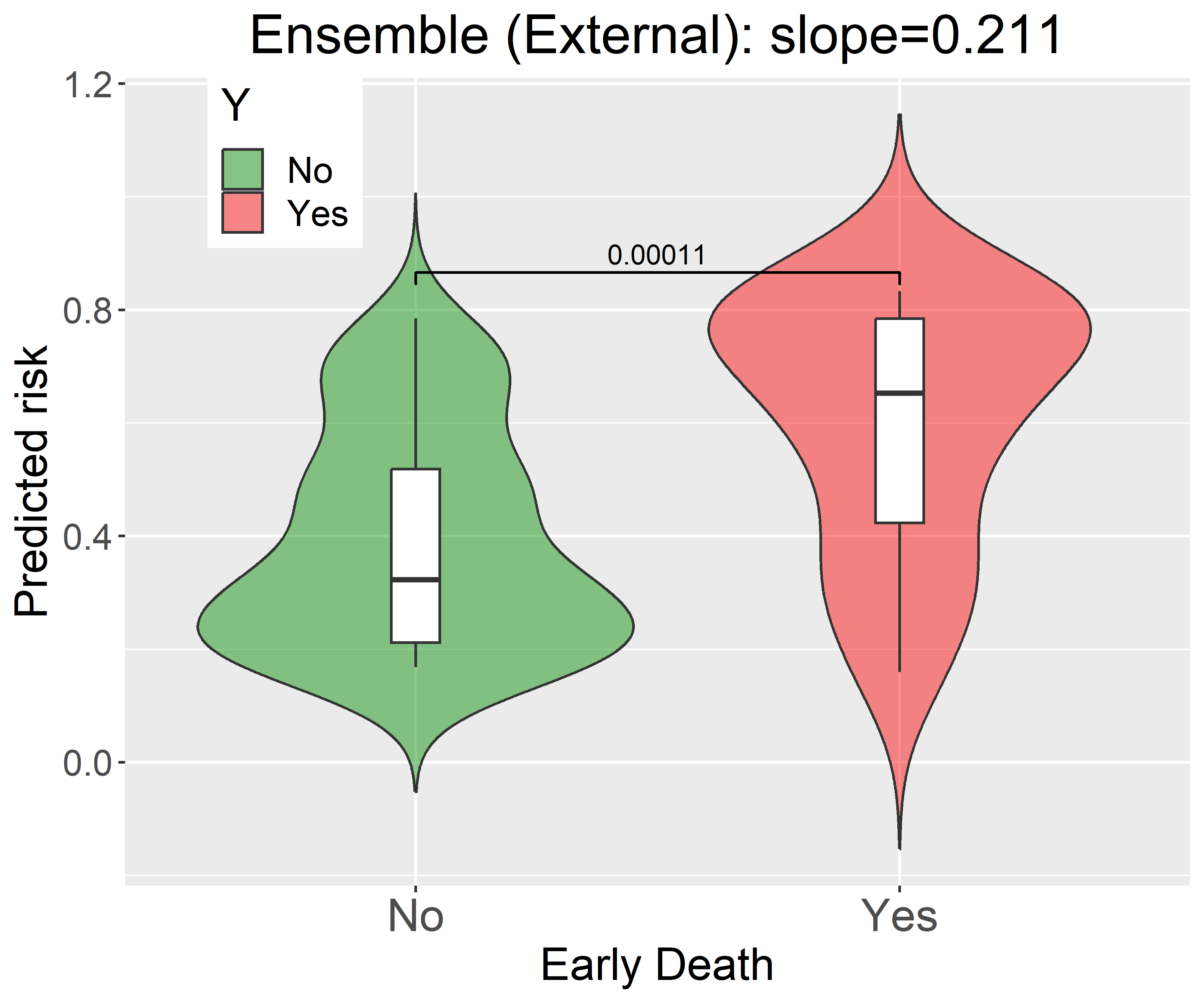

Supplement: Supplementary Figure 4 — Discrimination slope of the ensemble model in the external testing cohort. [file Image_4.tif]

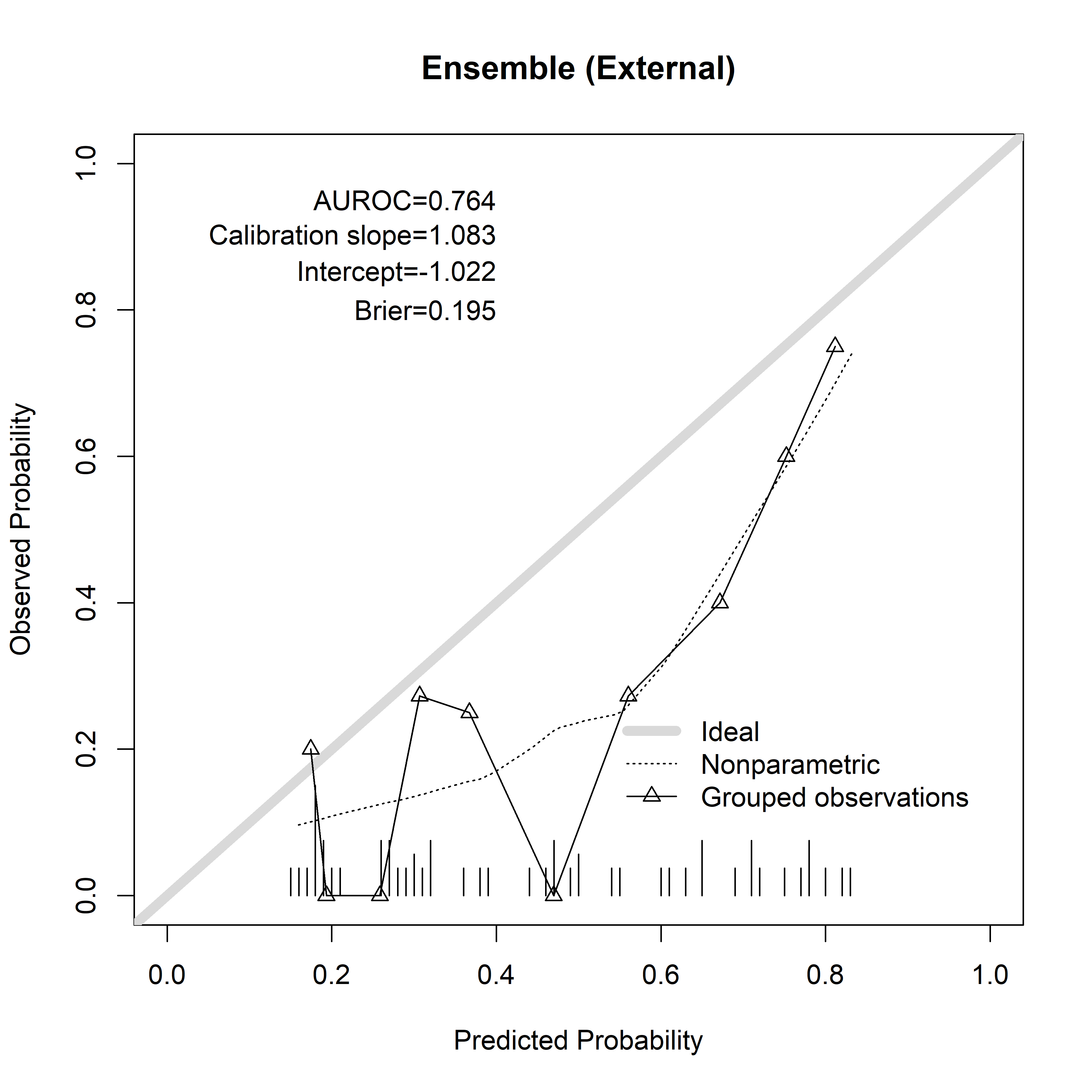

Supplement: Supplementary Figure 5 — Calibration plot of the ensemble model in the external testing cohort. [file Image_5.tif]

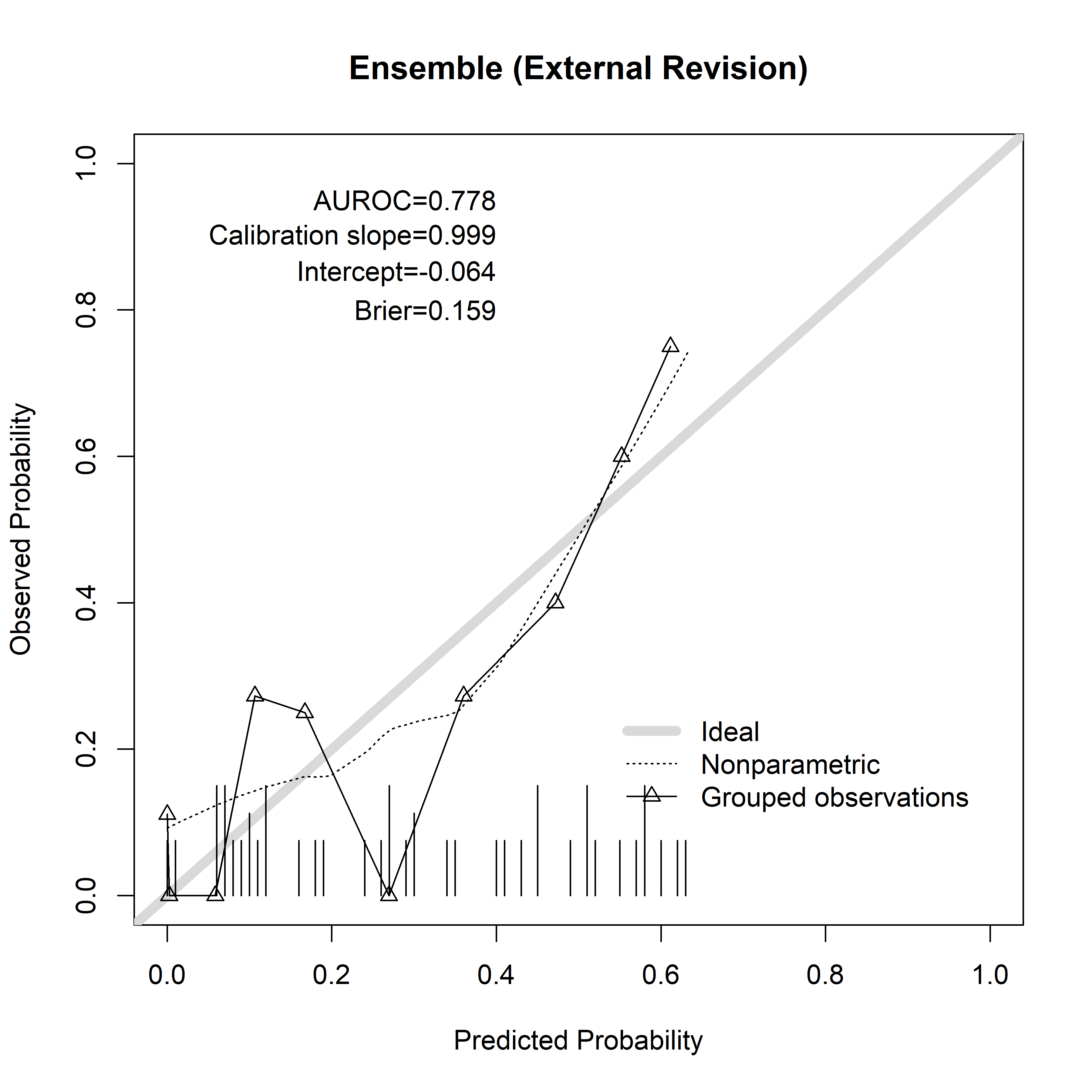

Supplement: Supplementary Figure 6 — Calibration plot of the ensemble model in the external testing cohort after model revision. [file Image_6.tif]

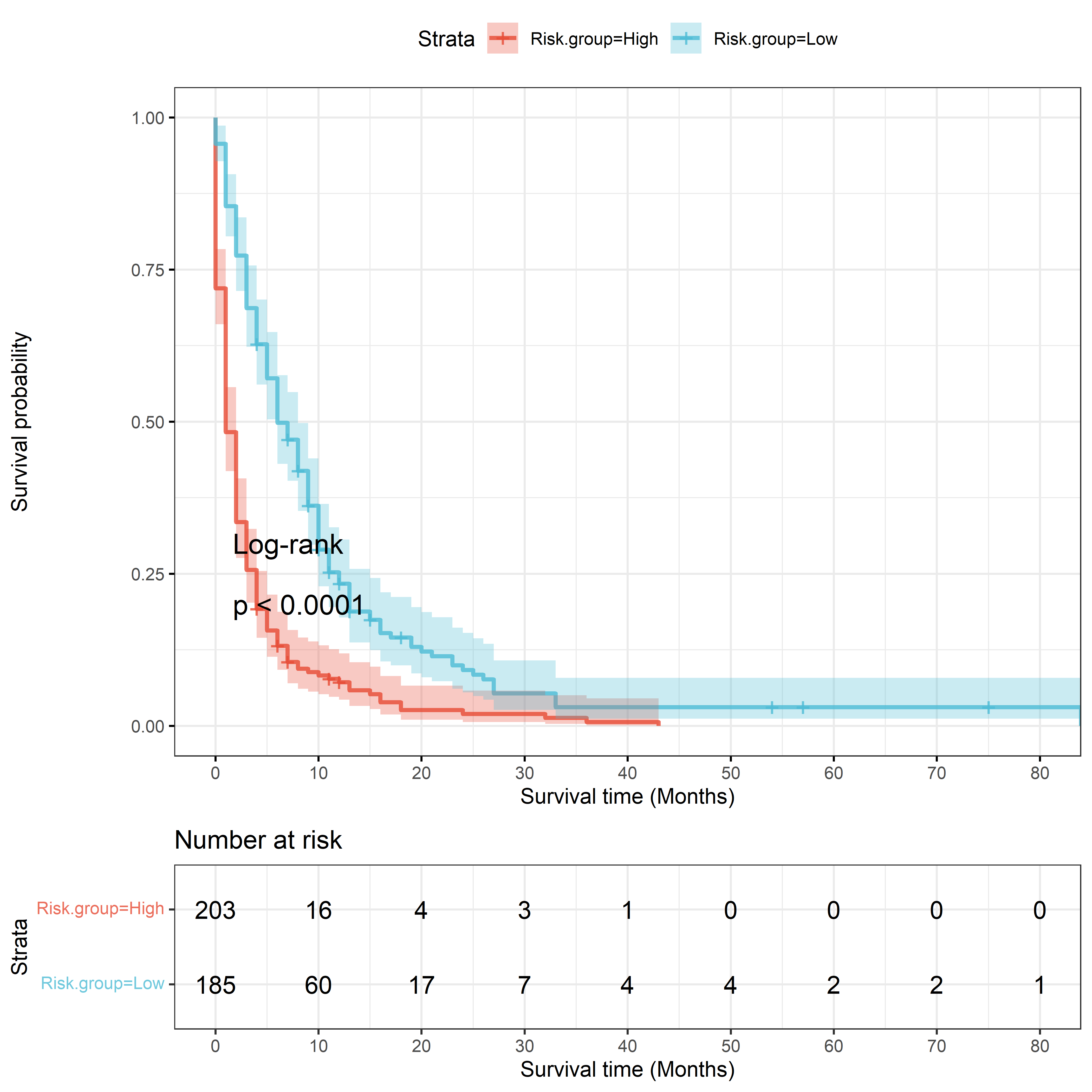

Supplement: Supplementary Figure 7 — Kaplan–Meier survival curve among patients stratified by risk group (p < 0.0001, log-rank test). [file Image_7.tif]
